# Whole-genome resequencing reveals positive selection and introgression signatures and genetic loci associated with early puberty traits in Chinese indigenous pigs

**DOI:** 10.1186/s12711-025-00975-1

**Published:** 2025-06-10

**Authors:** Minghao Cao, Tiantian Yuan, Dong Li, Yulong Wang, Lin Zhang, Jingchun Sun, Guangquan Lv, Rongrong Ding, Taiyong Yu

**Affiliations:** 1 Key Laboratory of Animal Genetics, Breeding and Reproduction of Shaanxi Province, Laboratory of Animal Fat Deposition and Muscle Development, College of Animal Science and Technology, Northwest A&F University, Yangling, 712100 China; 2 Key Laboratory of Agroecological Processes in Subtropical Region, Institute of Subtropical Agriculture, Chinese Academy of Sciences, Changsha, 410125 China; 3 Animal Breeding and Genomics, Wageningen University and Research, Wageningen, 6708PB The Netherlands

## Abstract

**Background:**

The genetic basis of the phenotypic diversity of pigs is regulated by variants across the genome, especially the trait of early puberty, which is a crucial trait for enhancing the reproductive ability of pigs and the economy of the pig industry. However, the genetic basis of the early puberty trait in pigs remains largely unknown.

**Results:**

Here, we report a comprehensive genomic variation map for pigs based on the resequencing of 493 accessions representing 59 different pig breeds or populations, which included 5,211,469 single-nucleotide polymorphisms (SNPs) and 487,725 small insertion/deletion structure variants (InDels). This sets included 45,640 high-quality structural variants (SVs). Our results suggested that Hanjiang black (HJB) pigs cluster with Jianghai-type pigs at the genetic level and that the genome characteristics of some HJB individuals exhibit a certain degree of European pig features. Using introgression and signature selection analysis, we identified several candidate genes associated with bone development and early puberty traits, such as *TBX5*, *PAPPA2*, *IGFBP3*, and *MKRN3*. Additionally, the GWAS and differential expression analysis results suggested that the *PAPPA2* gene is associated with early puberty in pigs.

**Conclusions:**

This study revealed that past introgression events could impact the agronomical traits of pigs and contribute raw material of genetics and breeding in pig. Moreover, our results suggest that the *PAPPA2* gene is a candidate gene associated with early sexual maturity in pigs and the genomic analysis provided important reference value for studying economic traits for pigs.

**Supplementary Information:**

The online version contains supplementary material available at 10.1186/s12711-025-00975-1.

## Background

Since the domestication of wild boars from Eurasia approximately 9000 years ago [1], natural selection and human-driven artificial selection have produced diverse phenotypic characteristics and genome structures of pig. Chinese indigenous pigs, such as the Laiwu black (LWB), Hanjiang black (HJB), and Bama xiang (BMX), exhibit remarkable characteristics in terms of meat quality, reproductive ability, and early puberty [2, 3]. For example, the BMX is an early puberty pig that reaches maturity at approximately three months, while European domestic pigs reach maturity at approximately five months [4].

Early puberty in pigs is defined as reaching puberty earlier by triggering gonadal maturation [5]. As an early phenotypic index of reproductive life [6, 7], the earlier age at puberty could increase reproductive life by producing more piglets to improve the financial benefit for the producer [8]. This trait involves multigene regulation and has medium heritability (*h*^2^ = 0.38–0.46) [9, 10] but the genetic basis of early puberty is still largely unclear. Using genome-wide association studies (GWAS) of SNP genotyping data, previous studies have identified several genes associated with early puberty, including *PRKD1* [11], *PAPPA* [12], and *OAS1* [13]. Although long-term studies of genetic basis about pig economic trait have been conducted [4], due to the limitations of individuals and breeds, few studies have analysed early puberty in pigs using whole genome sequence data.

It has long been argued that introgression can be a potential force in evolution and phenotype innovation [14, 15]. A growing body of evidence suggests a substantial role of introgression in past improvement of domesticated breeds [16, 17]. For example, the human-mediated introduction of Meishan (MS) pig germplasm improved the reproductive ability of European domestic populations during the 18th and early nineteenth centuries [16, 18]. The genetic basis of important economic traits could be revealed by detecting excess allele-sharing between breeds through high-throughput sequencing. However, the study of the impact of introgression from commercial to Chinese indigenous pigs is unclear and unprecise.

In this study, we collected germplasm from 493 pigs from 59 populations and sequenced them with an average depth of ~ 13.80 × . We reconstructed the phylogenetic relationships and genetic structures among all populations and assessed the genetic diversity and degree of genetic divergence among the different clades. Our results show what global excess allele-sharing between breeds has occurred between Eurasia populations. Moreover, GWAS and selection signal analysis revealed that a key gene is associated with early puberty, shedding light on the genetic basis of early puberty traits and providing essential insights for future pig breeding endeavours.

## Methods

### Sample collection and DNA extraction

We collected fresh ear tissue from 59 HJB pigs at the Heihe Pig Breeding Farm in Mian County, Shaanxi Province. We used standard cetyltrimethylammonium bromide (CTAB) to extract DNA [19] from the ear tissues. Libraries with an insert size of 300–500 bp were constructed, and paired-end reads (2 × 150 bp) were produced on a BGISEQ-T7 platform at BGI-Shenzhen, following the manufacturer’s procedures.

### Data collection and generation

Whole-genome sequencing (WGS) data for 434 pig samples was downloaded from the NCBI Sequence Read Archive (SRA, https://www.ncbi.nlm.nih.gov/sra/). Together with public data from previous studies (see Additional file 1: Table S1), the genetics variants panel used in this study included 124 European domestic pigs and 38 wild relatives, 247 Asian domestic pigs and 31 wild relatives, and 50 from North American domestic pigs. We also collected WGS from two Sumatra wild pigs and one warthog (*Phacochoerus africanus*) as an outgroup. Detailed data and geographic distributions for the sequenced pigs are shown in Additional file 1: Table S1.

### Short variation calling and annotation

Quality control of the raw sequencing data was performed using fastp (v.0.20.1) [20]. The high-quality reads were aligned to the pig reference genome (*Sus scrofa* 11.1) using Burrows-Wheeler Aligned (BWA) software (v.0.7.8) [21]. We then converted the mapping reads into bam files and sorted the files using SAMtools (v.1.10) [22]. Duplicates were removed by the MarkDuplicates module in GATK (v.4.2.6.1) [23]. Single-nucleotide polymorphisms (SNPs) and small insertions/deletions (InDels) were identified from the bam files by the GATK HaplotypeCaller module using the GATK best-practice recommendations [23]. Raw genomic variant call format (GVCFs) with the samples called individually were merged using GenomicsDBImport and converted for SNPs and InDels into variant files using GenotypeGVCFs [23]. We then selected the candidate SNPs and InDels and created the selected SNP and InDel dataset using the GATK module SelectVariants [23]. To avoid potential false-positive calls, we implemented “VariantFiltering” for the GATK selected SNPs and InDels using the best practice parameters “AC ≤ 0 | AF =  = AN | QUAL < 30.0 | QD < 2.0 | MQ < 40.0 | FS > 60.0 | SOR > 3.0 | MQRankSum < -12.5 | ReadPosRankSum < -8.0” and “AC ≤ 0 | AF =  = AN | QD < 2.0 | QUAL < 30.0 | FS > 200.0 | ReadPosRankSum < -20.0” [23]. We then filtered out non-biallelic SNPs and calculated the whole-genome sequencing coverage and depth of each sample using VCFtools (v. 0.1.16) [24] and SAMtools (v. 0.1.17) [22]. The sequencing depth of individuals ranged from 5.01 to 35.6, and the average, median, and standard deviation of depth were 13.8, 12.1, and 6.54, respectively.

### Structural variant calling

Structural variants (SVs) were identified from the sequence data of 292 high-depth samples (depth ≥ 10 ×) using Manta (v1.6.0) [25] with default parameters. We only retained deletions (DELs), duplications (DUPs), and insertions (INSs) that met the following two conditions: remain variants of the PASS and SVLEN labels and delete variants of the IMPREMISE label. The variants were merged using SURVIVOR (v1.0.7) [26], using a merge distance of 1000 bp, requiring type and no requiring strand to match, and 10% (292 * 10% approximately are 30) a minimum number of supporting caller. We then required all SVs to have a length between 50 and 100 Kb. Populations of the SV dataset include Asian domestic (AD), Asian wild pig (AW), Hanjiang black pig (HJB), European domestic pig (ED), American domestic pig (AmD), European wild pig (EW) populations. To annotate and predict the effects of the identified SNPs and InDels, we used SnpEff (v5.2) [27] to annotate the datasets, which filtered variants of allele count (AC) equal 0 and of the number of AC equal to allele number (AN). All subsequent analyses were performed using these data sets.

### Phylogenetic relationships and population structure, diversity, and divergence

To reveal the phylogenetic relationship between the Eurasian domestic pigs and their wild relatives, PLINK (v. 1.90) [28] was used to calculate the average shared allele distance matrix between individuals (–distance-matrix). The results of neighbor-joining (NJ) tree construction were displayed using MEGA11 [29] and iTOL [30]. Population structure was determined using the program ADMIXTURE (v. 1.3.0) [31]. The number of assumed genetic clusters K ranged from 2 to 30, and the best number of ancestral components was selected based on the minimum value of cross-validation (CV). Principal component analysis (PCA) was performed using PLINK and plotted using the R program, which calculated the top ten principal components.

To assess the genetic diversity among domestic Eurasia pigs and their wild relatives, we examined the patterns of pairwise linkage disequilibrium (LD) decay within each clade by random selection of approximately ten individuals from each clade, in order to avoid biases from differences in the number of individuals and breeds or populations. LD decay degree were measured as Pearson correlation coefficients (*r*^2^) using the PopLDdecay (v 3.41) software [32]. Distance when *r*^2^ dropped to half of the maximum value was represented the LD decay distance.

To estimate genetic diversity and degree of divergence among the clades, we calculated the distribution of pairwise fixation statistics (*F*_ST_) and nucleotide diversity (π) based on 50-Kb sliding windows with a 25-Kb step size using VCFtools (v. 0.1.16) [24].

### Quality of introgression

**Fig. 1:**
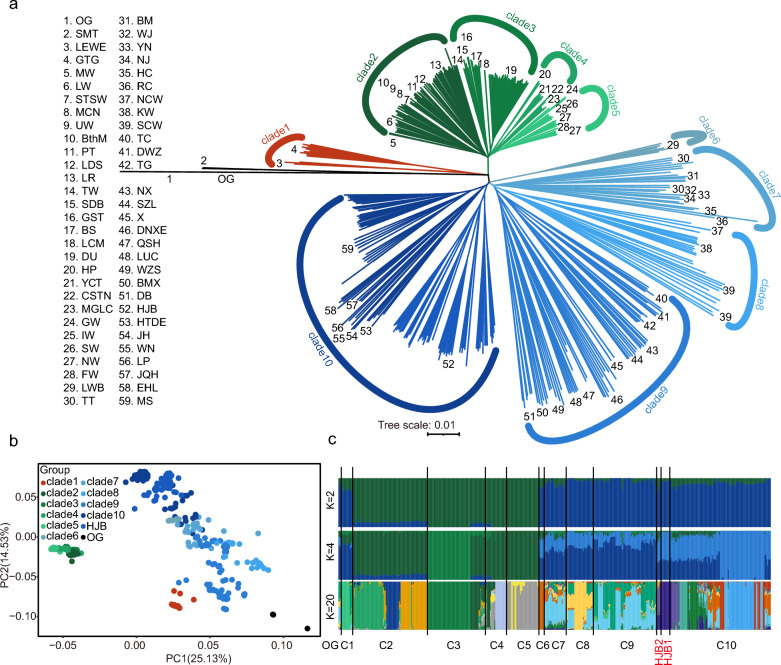
Phylogenetic relationships and population structure of 493 pigs based on SNPs. **a** Phylogenetic tree of 493 samples, including 14 wild progenitors and 53 domestic breeds. **b** Principal component analysis (PCA) plot of ten clades. **c** Ancestor component analyses with different numbers of K = 2, 4, and 20, in which C1-10 correspond to clade1-10, and HJB1 and HJB2 represent subgroups of HJB

To calculate excess allele-sharing among Eurasia domestic populations, we performed *F*-branch analysis to characterize the global introgression conditions, in which individuals who looked like potential F1 hybrids were selected, as indicated by close to 30/70 splitting in the admixture analysis, such as HJB2 (Fig. 1c). First, we selected 2 to 10 individuals from each clade and prepared a simplified version of the breed tree, which used vcf2phylip.py to convert variants calling formatting (vcf) file to nexus format and calculate the SVD score [33] using the PUAP* software to infer the breed phylogenetic tree based on the Dsuite common tutorial [34]. Dsuite was then run Dtrios, DtriosCombine, and finally the *F*-branch analysis, each time specifying the breed’s tree using the African warthog as the outgroup. Dsuite was used to first calculate *f* 4 value admixture ratios *f* (A, B, C, O) across the dataset, where combinations of taxa fit the necessary relationships ((A, B), C) in our phylogenetic tree. Significant instances of excess allele-sharing were identified by calculating a stringent Bonferroni multiple-testing significance threshold, which involved dividing the *P* value threshold of *P* < 0.01 by the number of cells in the *F*-branch matrix for which *f* b (C) could be calculated (646) and converting this to a *Z* score using dtools.py in Dsuite. All cells with *Z* scores higher than this threshold i.e., *Z* > 4.34 represented significant excess allele-sharing between taxa in the tree and were indicated as such.

### Localization of introgression

To localize the excess allele-sharing genes among clades, we confirmed function of these genes combined with Dinvestigate statistics. Dinvestigate is a model in Dsuite, which further analyses and explains the pattern of gene flow. It has three functions: (1) detecting the source of gene flow; (2) evaluating the direction of gene flow; (3) and calculating the ratio of gene flow. These analyses take advantage of a four-taxon statement (((P1, P2) P3) O). A negative D value indicates gene flow between P1 and P3, a positive D value indicates gene flow between P2 and P3, and D = 0 indicates no gene flow. We tested for the presence of interspecific genetic introgression from European domestics or wild relatives into Chinese indigenous and wild relative populations by different statistical analyses such as D statistics and *f* dm statistics using Dsuite [34]. We used HJB1 pigs as the reference population (P1), HJB2 pigs as the target population (P2), and LW pigs as the donor populations (P3). The Z score was calculated based on the standard errors at the whole genome level. A Z score greater than 3 (|*Z f* d|> 3) indicated a significant introgression region.

### Determining introgression directions

To identify the gene flow directions, the genome component of European commercial pigs’ transfer to the genome of Asian domestic pigs or reverse flow. We estimate gene flow direction using haplotype analysis and a phylogenetic tree based on the introgressed segment, which the criterion following: (1) from the genome segment of European commercial pigs to the genome of Asian domestic pigs, in phylogenetic tree, partly the Asian domestic populations nested within the European commercial clades, and in haplotype analysis, the haplotype of partly Asian domestic pigs same with European commercial pigs; (2) from the genome segment of Asian commercial pigs to the genome of European domestic pigs, in phylogenetic tree, partly the European domestic populations nested within the Asian commercial clades, and in haplotype analysis, the haplotype of partly European domestic pigs same with Asian commercial pigs; (3) mutual introgression with both scenarios[35].

### Detection of positively selected genes (PSGs)

The VCF file was separated by autosomes, and π ratio (π_late_puberty_/π_early_puberty_, as π_LP_/π_EP_) and *F*_ST_ was analysis to detect selected genes among early and late puberty populations (see Additional file 2: Table S2) with sliding windows of 50 Kb and a step size of 25 Kb using VCFtools (v0.1.16) [24]. XPCLR scores were also calculated using a 50 Kb sliding window with a step size of 25 Kb by XPCLR (v. 1.1.2) [36]. The top 5% of windows were considered to be candidate-selective regions influencing early puberty in pigs. After annotation with ANNOVAR [37], the sharing genes selected by using the three approaches, including *F*_ST_, π, and XPCLR, were considered to be positively selected genes (PSGs).

### Genome-wide association analysis of early puberty

To identify the genetic basis of early puberty in pigs, we used a mixed model approach implemented in EMMAX (v.20120210) [38], in which association mapping was performed using early and late puberty as a binary trait based on the characteristics of the germplasm of breed. Population includes 103 early and 103 late puberty pigs (see Additional file 2: Table S2). A strict Bonferroni multiple-testing *P* value threshold was calculated using the total number of SNPs tested: -log_10_ (0.05/1,351,664) = 7.43. To control the false positive ratio, we used the q value as a covariate when the CV error was lower based on the Admixture software. The significant sites were annotated using ANNOVAR [37].

### Annotation expression profiles of positively selected genes

To analyse the expression levels of the 124 PSGs, we downloaded the expression data (TPM) from the PIG RNA ATLAS database [39]. In total, approximately 80 categories of tissues/organs were included in the analysis. For each gene, the average TPM in approximately 80 categories of tissues/organs was calculated, and the genes whose TPM was at least 10 times the average TPM in a tissue/organ were considered “highly expressed genes”.

Gene Ontology (GO) and Kyoto Encyclopedia of Genes and Genomes (KEGG) analyses were performed using gProfiler [40] on the website (https://biit.cs.ut.ee/gprofiler/gost), and the results were plotted using the R program.

### RNA-seq analysis

To detect PSGs associated with early puberty, we performed an RNA-seq analysis of the pituitary. Samples from three individuals at early puberty (e.g., BMX) and three individuals at late puberty (e.g., LW) (see Additional file 3: Table S3) were retrieved from public databases. To process the transcriptome sequence data, we first used the SamToFastq tool of GATK software (v.4.2.6.1) [23] to convert the bam file into a fastq format file. Then, fastp was used for quality filtering of the sequencing reads. HISAT2 (v2.2.1) [41] was used for fast and accurate sequence alignment to *Sus scrofa* 11.1. Finally, a transcriptome gene expression count file was converted to obtain the gene expression profile in each sample using SAMtools (v1.15.1) and featureCounts (v2.0.3) [24, 42]. Differentially expressed genes were identified by DESeq2 (v.1.20) [43]. Genes with a corrected *P* value < 0.05 and fold changes > 2 or < 0.5 were considered significantly differentially expressed.

## Results

### Genomic variation map constructed

A total of 493 pigs from 59 populations around the world, including 18 European domestic and 6 wild relative, and 29 Asian domestic and 4 wild relative populations (see Additional file 1: Table S1), were collected to construct a pig variation map. After filtering, we identified a total of 5,211,469 high-quality SNPs, 487,725 InDels, and 45,640 SVs (Table 1). The average variant densities were 2248.5/Mb, 210.4/Mb, and 19.7/Mb for SNPs, InDels, and SVs, respectively. Among all SNPs and InDels predicted, respectively 282,311,086 and 38,425,424were effective sites, of which 2.69 (7,588,233) and 0.98% (377,012) were located in the predicted coding region.

**Table 1:** Summary of whole-genome variations identified in the 493 pigs

Variations	AD	HJB	AW	ED	AmD	EW	Total
SNPs	–	–	–	–	–	–	5,211,469
InDels	–	–	–	–	–	–	487,725
SVs	63,333	56,408	43,621	29,429	25,000	24,402	45,640
DELs	44,060	40,944	35,096	23,992	20,195	19,025	–
DUPs	3428	3577	2219	1290	1176	1277	–
INSs	15,845	11,887	6306	4147	3629	4100	–

SV number merged different groups among AD, HJB, AW, ED, AmD, and EW to exhibit a trend that the genome distribution difference from the different geographic regions. The total SVs number merged all germplasms based on one-time input to SURVIVOR software; thus, the total SVs number does not equal the SVs number added among groups based on geographic regions

We started from a total of 493 short-read WGS datasets with ≥ 5 × that were collected for the NCBI project (see Additional file 1: Table S1), of which 59.23% were kept after filtering, encompassing 292 (*Sus scrofa*) individuals. Based on the phylogenetic relationship (Fig. 1a) and geographical locations, we divided them into 6 main populations, including AD, AW, ED, EW, HJB, and AmD (Table 1 and see Additional file 4: Figure S1). Using an integrated SV calling pipeline, we discovered and genotyped a total of 45,640 high-quality SVs across 292 pigs. Across pigs, the SV count ranged from 1492 to 26,656, with a median count of 12,183. These non-redundant events included 35,443 DEL, 8065 INS, and 2132 DUP (Table 1 and see Additional file 4: Figure S1). Our results showed a trend in the SV distributions among Eurasia populations, with the number of SVs in Asian populations being greater than in European populations, and the number of SVs in HJB being intermediate within the Asian populations.

### Reconstructed phylogenetic relationships

To assess the phylogenetic relationships among Eurasia pig populations, we reconstructed a phylogenetic tree based on a high-quality whole-genome SNP dataset. HJB had a close relationship with Jianghai-type pigs, including MS, Erhualian (EHL), and Jiangquhai (JQH), which are originated from the Taihu region [44] in clade10. All breeds of South China pigs clustered in clade9, including BMX, Luchuan (LUC), and Diannanxiaoer (DNXE) pigs. The Korean wild (KW) pigs had a closer genetic distance to North China wild (NCW) than to the South China wild (SCW) in clade8, which could be caused by geographic proximity. Clade7 represents the Plateau type breeds, including Tibetan (TT), Wujin (WJ), and Bamei (BM), which live around plateau areas, such as the Qinghai, Tibetan, and Sichuan provinces. Interestingly, we found that the LWB pig independently clustered in clade6, and had a shorter genetic distance to other European pigs than Asian pigs, which may because introgression make similar of genomic component between Eurasian pig breeds [45]. In addition, the European pigs split into four clades, in which the domestic pigs from Britain clustered in clade2 and clade3, including Gloucester (GST), Yorkshire (LW), and Tamworth (TW) (Fig. 1a and see Additional file 1: Table S1). Their wild relatives were more closely related to the Yucatan (YCT), Mangalica (MGLC), and Casertana (CSTN) pigs. The Goettingen (GTG) and LEWE pigs, which are cultivated by Vietnamese Pot-bellied pigs, and the German domestic pigs [46] clustered in clade1.

The result of PCA was consistent with the phylogenetic tree (Fig. 1b). The results showed that PC1 separate the Eurasian pig populations. Along PC2, the Asian pigs were divided into Jianghai type pigs (clade10, deep blue), Plateau type pigs (clade7, sky blue), wild relatives of domestic pigs (clade8, bright sky blue), and South China type pigs (clade9, medium blue) (Fig. 1b). When assuming K = 2, ancestor composition analysis revealed that clades 2 to 5 are characteristic of European populations and clades 6 to10 are characteristic of Asian populations (Fig. 1c). When K = 4, the Duroc (DU) and other European populations were divided, as were the MS and other Asian populations (Fig. 1c), although there was no clear separation of domestic and wild relatives. When the K value equals 20, the CV error is lowest (see Additional file 5: Figure S2). And several HJB individual’s ancestor compositions are different compared with other one, in which HJB2 contains the ancestor compositions from European pigs (Fig. 1c).

### Genetic diversity and divergence analysis

**Fig. 2:**
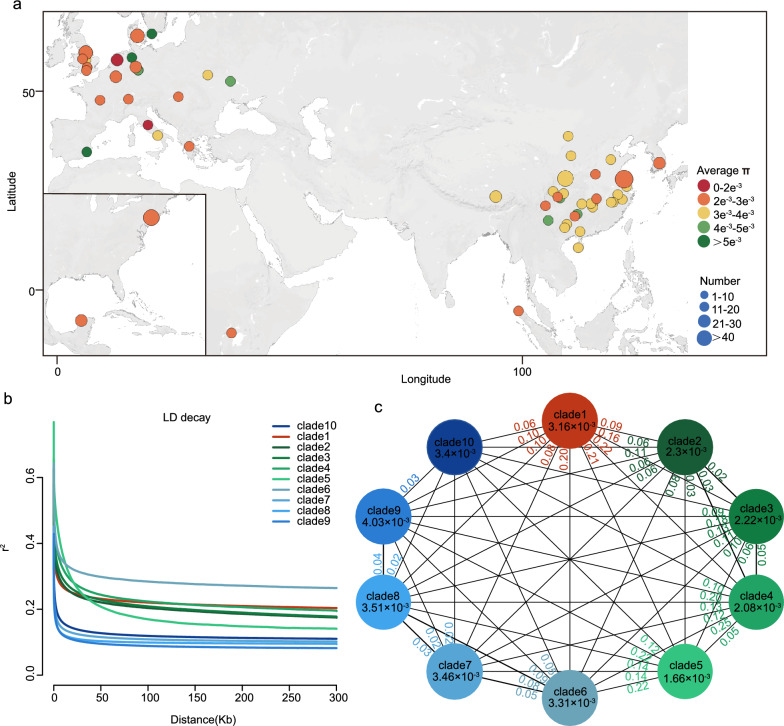
Genomic diversity and divergence between the ten clades. **a** Geographic distribution of 493 accessions from 59 breeds and populations and nucleotide diversity. **b** Clade-specific LD decay plots. **c** Nucleotide diversity (π) and genetic divergence (*F*_ST_) across the ten clades. The value in each circle is an estimate of π for each clade; values on each line indicate the *F*_ST_ between groups

To assess the genetic diversity of the worldwide pig populations, we calculated the nucleotide diversity (π) for each population. Asian populations generally had a high π value compared with European populations. However, there were also special cases, such as Linderodsvin (LDS) pigs (5.04 × 10^–3^) from southern Sweden, Bentheimer (BthM) pigs (5.09 × 10^–3^) from Britain, and Sattleschwein (STSW) (5.04 × 10^–3^) from Germany have greater π values than Chinese indigenous pigs, such as Jinhua (JH) (3.24 × 10^–3^) from Zhejiang Province, Jiangquhai (JQH) (2.58 × 10^–3^) from Jiangsu Province, and Xiang (X) (2.56 × 10^–3^) from Guangxi Province, China (Fig. 2a, see Additional file 1: Table S1, and Additional file 6: Table S4). To estimate the linkage disequilibrium decay (LD, measured as *r*^2^) patterns among different clades, we calculated the LD based on the SNP dataset. Clade4 (*r*^2^ = 0.34, 6.765 Kb) and clade5 (*r*^2^ = 0.40, 7.019 Kb), which included YCT, MGLC, CSTN, and some European wild pigs, had the longest decay distance. For the main European domestic populations (clade2 and clade3), the LD distance values ranged from 2.367 to 4.439 Kb and were generally greater than those for other Asian domestic breeds (0.286 to 0.807 Kb) (except clade6, because the distance more than 12 kb) (Fig. 2b and see Additional file 7: Table S5).

To quantify the genetic diversity and divergence between clades, we calculated π and the pairwise fixation indices (*F*_ST_) index. The π results indicated that clades 6 to 10, including the Asian pig populations, had a high genetic diversity (3.31 × 10^–3^-4.03 × 10^–3^) compared to clades 2 to 5 (1.66 × 10^–3^-2.3 × 10^–3^), including the European pig populations (Fig. 2c), which is consistent with the nucleotide diversity of each population (Fig. 2a). Moreover, the *F*_ST_ results showed that the average level of genetic divergence among the European pig populations was approximately 0.04, ranging from 0.02 to 0.15. Similarly, the average *F*_ST_ value among the Asian populations was approximately 0.043 (Fig. 2c). Interestingly, clade6, containing LWB, had a high *F*_ST_ value (0.05 ≤ *F*_ST_ ≤ 0.09) compared with other Asian clades. Moreover, there was a high level of genetic divergence between Eurasian populations (average *F*_ST_ = 0.13) (Fig. 2c).

### Genetic contribution of commercial relatives to Asian pigs

**Fig. 3:**
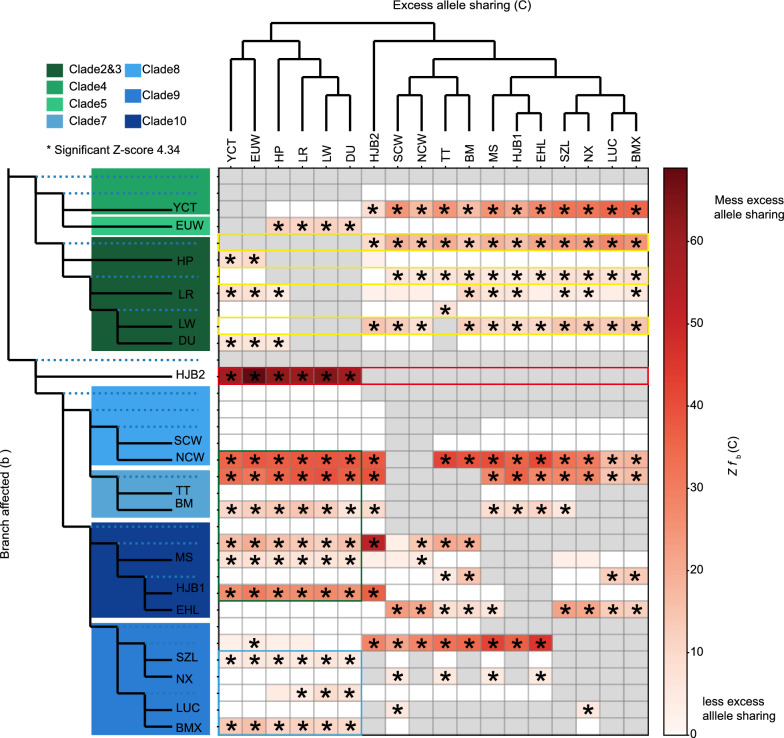
Global excess allele-sharing evaluation. **a** *F*-branch (*f* b(C)) statistics across our dataset highlight excess allele-sharing between Eurasia pigs. The redness of each cell in the matrix indicates the degree of excess allele-sharing of each tree tip (C) with each tip or node **b** with significant instances of excess allele-sharing, where a *Z* score > 4.34 (equivalent to the Bonferroni multiple-testing corrected *P* value of 0.01) is highlighted with a star. The red box represents the excess allele-sharing between HJB2 and other populations; the green and blue box represents the excess allele-sharing between Asian and European populations; the yellow box represents the excess allele-sharing between European pigs and other Asian pigs

**Fig. 4:**
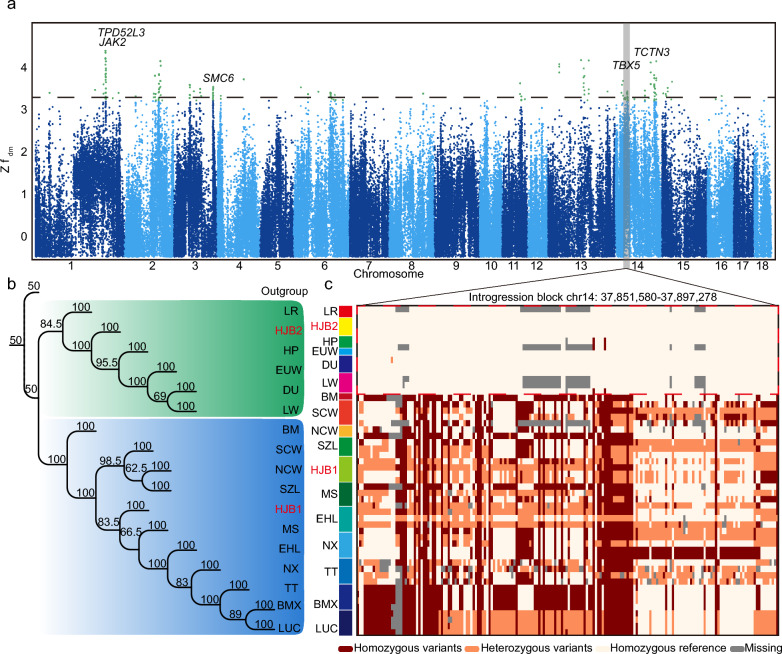
Potential excess allele-sharing signals from LW into HJB. **a** Manhattan plot of values showing the introgression signals from LW to HJB based on the Z (*f* dm) value. **b** Phylogenetic relationships of the haplotypes within the introgression block region according to breed information see Additional file 8: Table S6. **c** Two haplotype patterns inside the haplotype block of the *TBX5* gene. Rows represent haplotypes sorted by type and population. Columns denote variants from position chr14: 37,851,580 to 37,897,278 (the quality control is MAF = 0.35). The lattices in red, orange, and white indicate homozygous variants, heterozygous variants, and heterozygous reference alleles, respectively

Admixture analysis revealed genetic components from European commercial breeds in several Chinese indigenous pig populations (Fig. 1c). Excess allele-sharing between breeds and populations across our dataset (see Additional file 8: Table S6) reflected significant introgression between European and Asian pigs (Fig. 3, the star mean is significant *Z f* b(C) > 4.34). Our results suggest that the South China type pigs (Fig. 3, blue box), such as Shaziling (SZL), Ningxiang (NX), and LUC, were less genetically admixed with alleles from European commercial pigs than Jianghai type pigs and their wild relatives (Fig. 3, green box). In addition, ancestor component in clades 2 and 3 had contributed to genomic component of Asian domestic pigs, (Fig. 3, yellow box). Moreover, genomic component of several HJB individuals (Fig. 1c, such as HJB2) were significantly mixed with alleles from DU, LW, and LR (Fig. 3, red box). Our results indicate significant introgression signatures, with the peak signature including the *TBX5* gene (Fig. 4a, and see Additional file 9: Table S7, Additional file 10: Table S8, and Additional file 11: Table S9). The phylogenetic tree and the haplotype of the introgression block (Fig. 4b, c) revealed that HJB populations were divided into two clades, in which HJB1 had a mosaic haplotype that was consistent with those of other Chinese indigenous pigs. At the same time, HJB2 shared the homozygous reference haplotype that was consistent with European commercial pigs (Fig. 4b, c). The different haplotypes may be mean potential of different functions in the gene region of *TBX5* between HJB1 and HJB2, which may impact the phenotype of some HJB individuals due to introgression, while not influencing other pigs (HJB1).

### Identification of positively selected genes

**Fig. 5:**
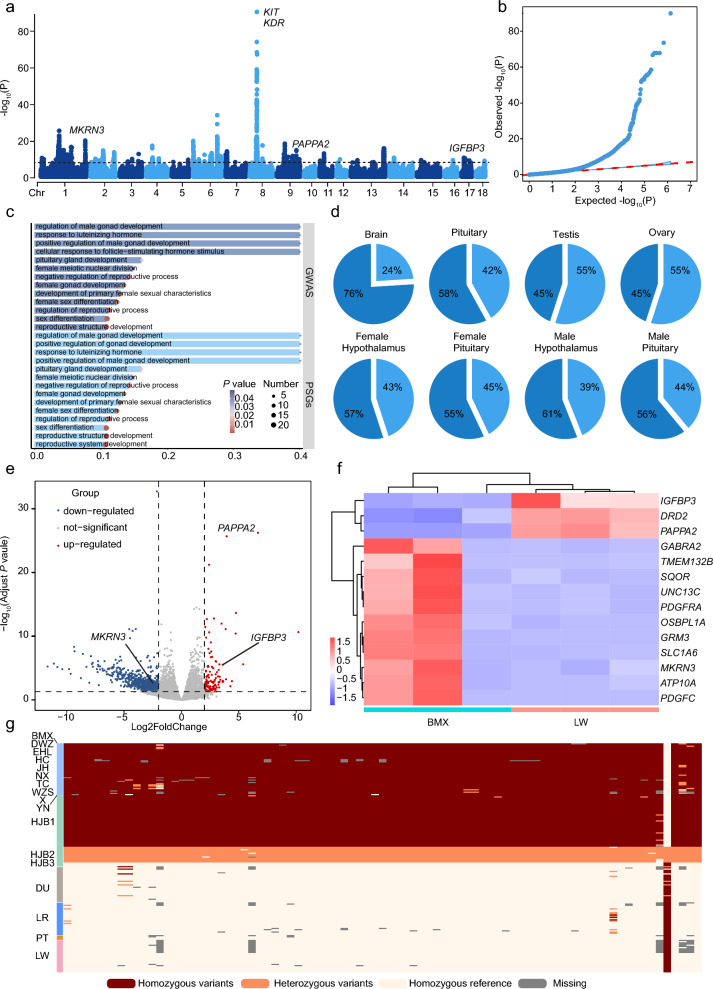
Positively selected genes (PSGs) associated with early puberty. **a**, **b** Manhattan plots of cc-GWAS between early and late populations and a QQ plot. **c** Enrichment analysis of genes between PSGs and GWAS. **d** The proportions of highly expressed genes in the 176 gene that were identified as PSGs and using GWAS were disproportionally enriched in differentially expressed tissues according to the gene expression data from the Pig ATLAs. **e** Volcano plot of differentially expressed genes (DEGs) in pituitary tissues between LW and BMX at 85 days. **f** Heatmap showing the changes in the expression of the 14 common DEGs in the pituitary. **g** The haplotype of genomic regions at the *PAPPA2* site between early and late puberty pigs

To detect selection signatures associated with early puberty, we scanned the genome of the early versus late puberty populations using three methods, namely the *F*_ST_, nucleotide diversity (π) ratio, and XPCLR, (see Additional file 2: Table S2). The top 5% ranked genes based on each strategy were considered associated with early puberty pigs. The top 5% of the ranked windows based on the π ratio (π_EP_/π_LP_), *F*_ST_, and XPCLR (see Additional file 12: Figure S3 a-c) contained 2141, 1893, and 3292 genes, respectively (see Additional file 13: Table S10, Additional file 14: Table S11, and Additional file 15: Table S12). Gene Ontology (GO) analysis revealed that these genes were mostly enriched for terms related to the reproductive system that are associated with the function of sexual system development (Fig. 5c, and see Additional file 16: Table S13, Additional file 17: Table S14, and Additional file 18: Table S15).

To avoid a false positive ratio, genes that were shared within the top 5% based on the three approaches were identifies as PSGs, which resulted in467 PSGs (see Additional file 12: Figure S3 d and Additional file 19: Table S16). GO analysis revealed that more functional terms associated with sex were enriched among these PSGs, indicating that PSGs play an important role in early puberty in pigs (Fig. 5c). Our results also revealed that the PSGs were enriched for terms related to the nervous system and sex hormone regulation, including “regulation of intracellular estrogen receptor signalling pathway” (GO:0033146), “response to estrogen” (GO:0043627), and “pituitary gland development” (GO:0021983) (see Additional file 20: Table S17). We then hypothesized that PSGs were more likely affected early puberty in pigs via the HPG axis.

### PSGs closely related to the early puberty trait

To identify PSGs associated with the early puberty trait, we performed case–control genome-wide association studies (cc-GWAS). The GWAS revealed 1567 SNPs (see Additional file 21: Table S18) in the whole genome region that reached genome-wide significance at a Bonferroni-corrected threshold of *P* < 3.7 × 10^–8^. These regions included 1275 genes (see Additional file 22: Table S19), including 176 genes (see Additional file 23: Table S20) that overlapped those from the *F*_ST_, π ratio, and XPCLR analyses. We found that the regions on chromosome 1 (*MKRN3*) (*P* = 2.59 × 10^–8^) and chromosome 9 (*PAPPA2*) (*P* = 1.13 × 10^–10^) were strongly associated with early puberty (Fig. 5a, b, and Additional file 21: Table S18). The GO enrichment results for these 176 genes revealed that terms related to reproductive and sexual development, including “reproductive system development” (GO:0061458), “sexual reproduction” (GO:0019953), and “development of primary sexual characteristics” (GO:0045137) (Fig. 5c and see Additional file 24: Table S21).

To further investigate the functions of genes that were identified as between PSGs and cc-GWAS, we searched the expression profiles of the 176 genes in the PIG RNA ATLAS [39] database, which included data on 164 of the 176 genes (see Additional file 25: Table S22). Among these 164 genes, 125 (76.2%) were highly expressed (at least tenfold of the average TPM) in the brain, while 95 (57.9%) were highly expressed in the pituitary. In addition, 74 (45.1%) of these genes were highly expressed in both testis or ovary (Fig. 5d and see Additional file 25: Table S22). Next, we compared the expression of all genes among subregions of the brain between males and females and found that the number of highly expressed genes for males was greater in the hypothalamus (61.0%) than in the pituitary gland (56.1%) (Fig. 5d and see Additional file 26: Table S23); however, in females, the number of highly expressed genes was greater in the pituitary (56.7%) than in the hypothalamus (54.9%) in female (Fig. 5d and see Additional file 27: Table S24). Our results showed that all sharing genes may play an important role in HPG axis modulating early puberty.

To investigate the expression of the PSGs in pituitary tissue between early and late puberty, at 85 days, we conducted a transcriptomic analysis of both early (BMX) and late (LW) pigs RNA-seq data from CRA000876 (see Additional file 28: Figure S4 a, and Additional file 3: Table S3). Data on total of 13,342 genes were obtained from the RNA-seq analysis between BMX and LW pigs. After filtering, we found 865 differentially expressed genes (DEGs), of which 127 were upregulated and 738 downregulated (Fig. 5e and see Additional file 29: Table S25). The set of intersection of the PSGs and the cc-GWAS genes included 14 DEGs in the pituitary tissue (Fig. 5f). GO analysis revealed that these genes were significantly enriched for terms related to sexual characteristic development and sex differentiation (see Additional file 28: Figure S4 b and Additional file 30: Table S26). Finally, we retained the 11 genes that overlapped between the PSGs, the genes identified via GWAS, and the DEGs as genes that may play important roles in regulating precocious puberty in pigs, including *IGFBP3*, *PAPPA2*, and *MKRN3* (Fig. 5a and f). Notably, one of these candidate genes, *MKRN3*, is a star gene associated with human central precocious puberty [47], which proved that the overlapping genes (Fig. 5f) may have other potential gene with early puberty. The SNP haplotype of the *MKRN3* gene differed between the early and late puberty populations. The early puberty individuals (e.g., BMX, NX, and HJB) [2, 4] had homozygous variant haplotypes, in which homozygous variants haplotype mean the two allele for each loci differ with reference genome in all region of *MKRN3* gene; however, late puberty individuals harboured characteristics of homozygous reference haplotypes, in which homozygous reference haplotypes mean the two allele for each loci same with reference genome in all region of *MKRN3* gene, such as LW and DU (see Additional file 31: Figure S5) [11, 48].

Another important gene, *PAPPA2*, was found to have undergone strong positive selection (Fig. 5a and see Additional file 12: Figure S3) and exhibited elevated expression in the pituitary tissue of late puberty pigs (Fig. 5e, f). Combined with haplotype analysis (Fig. 5g), the *PAPPA2* gene was identified as a key candidate gene associated with early puberty. However, the mechanism by which this gene regulates precocious puberty requires further investigation.

## Discussion

There are approximately one hundred breeds or populations of pigs in China [49], which can be divided in six groups based on geographical distributions, including Central China type, South China type, Southwest China type, Jianghai type, North China type, and Plateau type [2]. Previous studies [50–52] have reconstructed the phylogenetic relationships of Eurasian pigs, and divided them into four to six groups for Chinese domestic populations, consistent with similar phylogenetic relationships (Fig. 1a) identified in this study based on whole genome sequencing data. Notably, the Mangalica (MGLC) pigs from Hungary, Casertana (CSTN) pigs from south Italy, and Yucatan (YCT) pigs from Mexico presented greater genetic distances from other pigs originated from European (Fig. 1a) because the MGLC has a greater degree of genetic divergence (0.46 < *F*_ST_ < 0.52) than other European domestic pig populations [53]. These findings provide evidence for the correct phylogenetic relationships of Eurasians. Moreover, we found that Jianghai type (clade10) pigs, including MS, EHL, and HJB, have a special ancestral component, consistent with previous results [54], which may represent Liangchu culture domestication 4000 to 5000 years ago[55].

### Genetic diversity and divergence analysis

High genetic diversity can improve a population’s ability to adapt to climate change and provide breeding materials to meet increasing human demands for food [56]. The genetic diversity results represent only a trend because our estimates of nucleotide diversity (π) are biased downward due to excluding all missed SNPs that missing genotype or not detected variants. Our results revealed a relationship between nucleotide diversity and the short LD decay distance in clades of high genetic diversity. For example, European commercial pigs have low genetic diversity and longer LD decay distances, while Asian domestic pigs have shorter LD decay distances. This trend has also been found in other livestock species, such as sheep [57], goat [58], and cattle [59]. The degree of genetic divergence measures the similarity of the genome, and *F*_ST_ values generally correlate with geographic distance [60]. Our results show that the *F*_ST_ value within European or Asian populations is small, which is consistent with previous results [16]. However, LWB appeared to have a higher *F*_ST_ value than other Asian populations, which may be because LWB has undergone introgression from European commercial pig breeds [45]. In recent years, genetic divergence has been assessed using microsatellites [61], SNP genotyping data [62, 63], and whole genome sequences [16], in which microsatellites used in early stage, and SNP genotyping data present later, and WGS was used to estimate genetic diversity in recent decade. Moreover, the genetic markers in microsatellites and SNP genotyping data are few and far between genomes. And WGS data may more present reality condition of genetic diversity. Because of the lack of genetic marker density and breed information, more genome data, such as WGS, long-read sequence, and long-read transcriptomics, may be required to obtain to precise information on differences of genomic sequence reflected genetic diversity.

### The influence of European commercial breeds on HJB genomes

Introgression events during the Industrial Revolution between Eurasia pigs has widely reported [54, 64, 65], Chinese breeds were imported to Europe to improve commercial traits in European breeds [16]. Actually, any introgression signatures may be widespread, but the introgressed haplotypes are rarely fixed in hybrids pigs’ genome. The Asian introgressed haplotypes are associated with regions that harbour genes involved in meat quality, growth rate, and fertility[16]. Recent study of global introgression from commercial to Chinese indigenous pigs is unclear for reveal economic trait in pig. In this study, the HJB2 was to have a significant *f* b(C) (|*Z f* b(C)|> 40) with commercial pigs, such as DU, LW, and LR pigs and detected excess allele-sharing blocks between some HJB individuals and LWs. The *TBX5* gene is more important for limb [66] and heart [67] development and is associated with animal welfare, for example, osteochondrosis [68], in the pig industry. The LW pig is a commercial breed that suffers from osteochondrosis [69, 70]. If the HJB population harbours the same haplotype as the LW population for some beneficial genes, we should focus on maintain these individuals and apply breeding the next generation.

We also focus on other introgression peaks on other chromosomes. For example, the *TPD52L3* gene is located on chromosome 1 and some studies have indicated a potential role for *TPD52L3* in testis development and spermatogenesis [71]. The *JAK2* gene, as a introgression gene (Fig. 4a), which participates in JAK2-STAT5 signalling pathways, is not sensitive to the porcine growth hormone (pGH) in young pigs, which explains why the growth of neonatal pigs is GH independent [72]. The *JAK2* gene has also been mapped to a suggestive QTL region in a Berkshire x Yorkshire cross for loin eye area (*P* = 0.009), fatness, colour, and pH [73]. Interestingly, we also found the *TCTN3* on chromosome 14, as a introgression gene (Fig. 4a), another peak that has been associated with litter traits of pigs [74]. On other peak such as *SMC6* located on chromosome 3 (Fig. 4a), which is associated with congenital splay leg [75]. In summary, these methods above could increase the ability to mine key genes for remarkable traits and may accelerate a process of precise breeding. Thus, detection of introgression signatures may be an important approach to detect candidate genes for important traits in domestic animals.

### The genetic basis of early puberty

Early puberty in pigs is defined as a shorter time to reach sexual maturity, for which a genetic basis and several genes, such as *AHR*, *ANKRA2*, and *FRS2* [4], have been identified. However, these studies are still limitation because of far and rare the number of genetic variants, or insufficient omics analysis. To avoid false positives, we intersected the gene sets from three approaches as PSGs associated with early puberty. The identified functions of the PSG are related to regulation of sex hormone secretion and development of reproductive organs, which may indicate a relationship between PSGs and early puberty. Case–control whole genome association study identified the *KIT* gene, which influences coat colour [76], and the *KDR* gene, which regulates the content of intramuscular fat [77]. To precisely locate the regulated gene of early puberty traits, we also intersected between the PSGs and gene set of cc-GWAS, and annotated intersected gene set expression based on the Pig ATLAs database [39]. And result show that the PSGs were found to mainly influence the brains or pituitary of early puberty pigs. In the brain subregions, we found that, in males, the ratio of genes with high expression in the hypothalamus was greater than that in the pituitary tissue, while the opposite was observed for females. We hypothesize that tissue regulation during early puberty involves the hypothalamus in males and the pituitary in females. For example, Shi et al. [5] showed that genetic ablation of the *TBX3* gene in hypothalamic KNDy neurons in mice resulted in a marked delay in male puberty onset, although the mice failed to reach sexual maturation, as determined by the lack of ovulation; however, female knock-out mice exhibited only a subtle delay in the timing of vaginal opening [5]. Thus, we next performed transcriptome analysis using pituitary tissue data to identify difference expression gene of modulation early puberty. We then intersected the identified gene sets based on DEGs, GWAS, and PSGs, which identified the *MKRN3*, *IGFBP3*, and *PAPPA2* genes. The *MKRN3* [47] and *IGFBP3* [78] genes have been widely reported to regulate early puberty in humans. As described above, the *TBX3* gene not only is a classical gene that influences body size [79] but also plays a key role in regulation of early puberty [5]. The function of the *PAPPA2* gene has been widely reported as a protease, whose function loss may increase the concentrations of circulating IGFBP5 and IGF-I and decrease the concentration of circulating IGFBP3 [80]. An increase in IGFBP levels may be related to follicular atresia [81], further impacting the estrogen release and early puberty. In addition, increasing the concentration of IGF-I led to girls reaching puberty more quickly [82]. On the other hand, the *PAPPA2* gene is closely related to bone development [83] and body size [84]. For example, girls who are taller or shorter in body size reach puberty earlier [82] than average size girls. Our results revealed that the *PAPPA2* expression is significantly up-regulated in pituitary tissue of late puberty populations, further emphasizing the role of this gene in early puberty. However, there is still a lack of evidence showing that the *PAPPA2* gene directly regulates early puberty in pigs, and more solid evidences are needed to test this hypothesis.

## Conclusions

In summary, we reconstructed the phylogenetic relationships and genetic structures among our investigated populations, and quantified their genetic diversity and the degree of genetic divergence among different clades. Our results showed that global introgression has occurred between Eurasian populations. In addition, GWAS and selection signature analysis revealed that the *PAPPA2* gene is associated with early puberty in Eurasian pigs, shedding light on the genetic basis of early puberty traits and providing essential insights for future pig breeding endeavours.

## Supplementary Information

Additional file 1: Table S1. Description of the 493 pigs used in this study.

Additional file 2: Table S2. The population information of the early and late puberty to conduct selection signal analysis.

Additional file 3: Table S3. The RNA-seq data information between BMX and LW.

Additional file 4: Figure S1. The SV distribution for each individual and different group.

Additional file 5: Figure S2. The admixture results in K from 2 to 30, except 2, 4, and 20, and the cross-validation scatter plot.

Additional file 6: Table S4. Nucleotide diversity value among different pig breeds or populations.

Additional file 7: Table S5. Linkage disequilibrium decay distance among different pig clades.

Additional file 8: Table S6. The population information for F-branch analysis.

Additional file 9: Table S7. The information of significant loci associated with introgression loci.

Additional file 10: Table S8. Genes located in significant introgression block in HJB2 and commercial pigs.

Additional file 11: Table S9. Functional categories of the genes located in significant introgression block in HJB2 and commercial pigs.

Additional file 12: Figure S3. Venn diagram of three selection signature approaches and shared gene sets. a. The π ratio result is plotted in the Manhattan plot. b. The *F*_ST_ result plotted the Manhattan plot. c. The XPCLR result plotted the Manhattan plot. d. The Venn plot showed the sharing gene sets in all of the methods.

Additional file 13: Table S10. Genes located in the top 5% high nucleotide diversity regions in early puberty pig as compared to late puberty pigs.

Additional file 14: Table S11. Genes located in top 5% FST regions in early puberty pig as compared to late puberty pig.

Additional file 15: Table S12. Genes with top 5% XP-CLR score in early puberty pig as compared to late puberty pig.

Additional file 16: Table S13. Functional categories of the genes located in the top 5% high nucleotide diversity regions in early puberty pigs as compared to late puberty pigs.

Additional file 17: Table S14. Functional categories of the genes located in the top 5% FST regions in early puberty pigs as compared to late puberty pigs.

Additional file 18: Table S15. Functional categories of the genes with top 5% XP-CLR score in early puberty pigs as compared to late puberty pigs.

Additional file 19: Table S16. Sharing 467 positively selected genes (PSGs) in early puberty pig as compared to late puberty pig.

Additional file 20: Table S17. Functional categories of the genes with sharing 467 positive selected genes (PSGs) in early puberty pigs as compared to late puberty pigs.

Additional file 21: Table S18. The information of significant loci associated with early puberty using emmax software.

Additional file 22: Table S19. Genes identified using emmax software between early puberty and late puberty pig.

Additional file 23: Table S20. The sharing sets of genes between PSGs and GWAS genes.

Additional file 24: Table S21. Functional categories of the genes identified using emmax between early puberty and late puberty pigs.

Additional file 25: Table S22. The expression level of genes using the PigATLAs database annotated among different tissues for the sharing set of genes between PSGs and GWAS genes.

Additional file 26: Table S23. The expression level of genes using PigATLAs database annotate among male brain subregion for the sharing set of genes between PSGs and GWAS genes.

Additional file 27: Figure S24. The expression level of genes using PigATLAs database annotate among female brain subregion for the sharing set of genes between PSGs and GWAS genes.

Additional file 28: Figure S4. Transcriptome analysis result. a. PCA plot of transcriptome analysis of pituitary tissues in the LW and BMX groups at 85 days. b. GO enrichment analysis of the DEGs between LW and BMX.

Additional file 29: Table S25. Differentially expressed genes (DEGs) between BMX and LW.

Additional file 30: Table S26. Functional categories of the genes with differentially expressed genes (DEGs) between BMX and LW.

Additional file 31: The haplotype plot of genomic regions at the *MKRN3* gene in early and late puberty pigs.

## Data Availability

Raw sequencing data that support the findings of this study have been deposited in the NCBI BioProject database under the list (see Additional file 1: Table S1). The sequencing data of 59 HJB individuals used in this study have been submitted to the National Centre for Biotechnology Information (NCBI) with the accession number PRJNA1035607. The RNA-seq data of the pituitary were collected from the Genome Sequence Archive (GSA) with the accession number CRA000876.
