# Supplementary figures and images for: Whole-genome resequencing reveals positive selection and introgression signatures and genetic loci associated with early puberty traits in Chinese indigenous pigs
Source: Genet Sel Evol. 2025 Jun 10;57:29. doi: 10.1186/s12711-025-00975-1 (PMC12150574; doi:10.1186/s12711-025-00975-1)

SV distribution for each individual

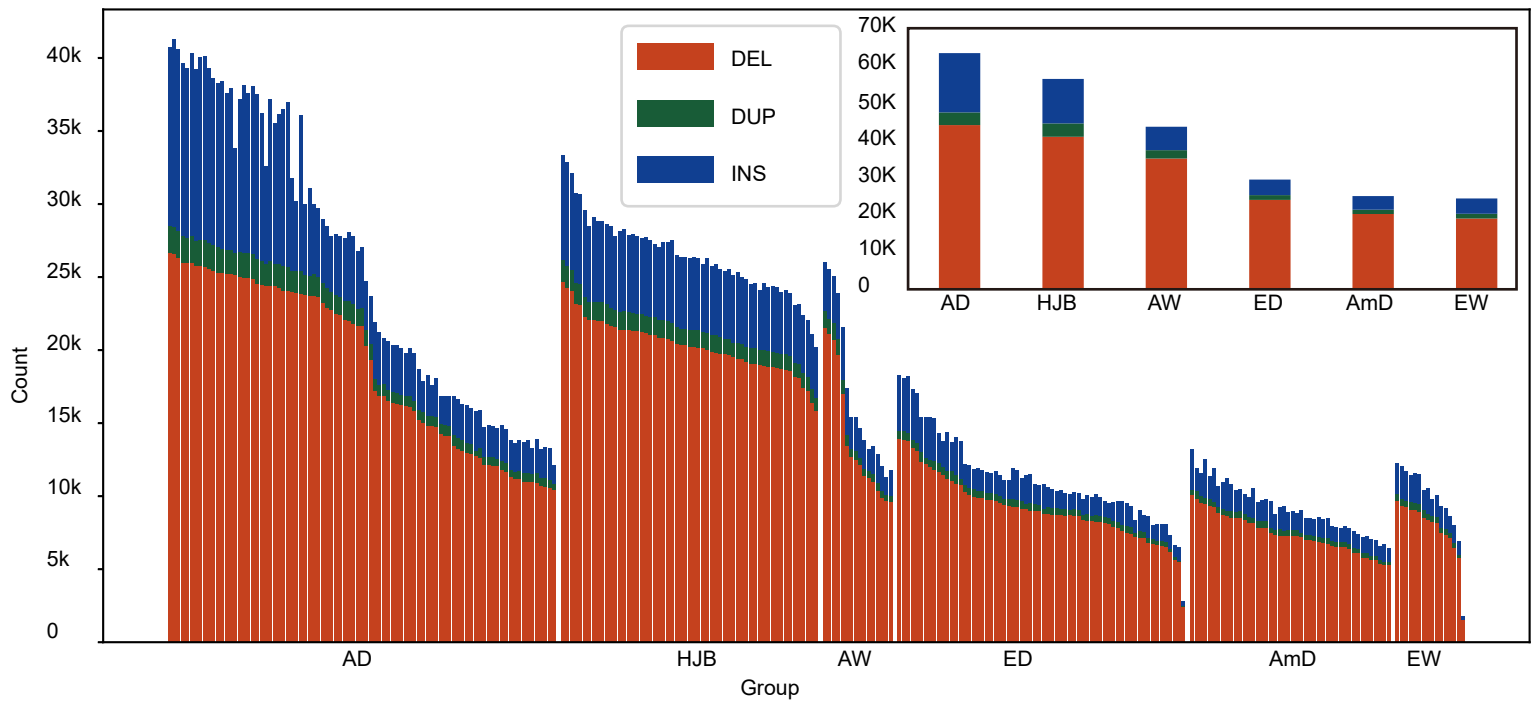

Supplement: Supplementary file 4 — Additional file 4: Figure S1. The SV distribution for each individual and different group. [file 12711_2025_975_MOESM4_ESM.pdf]

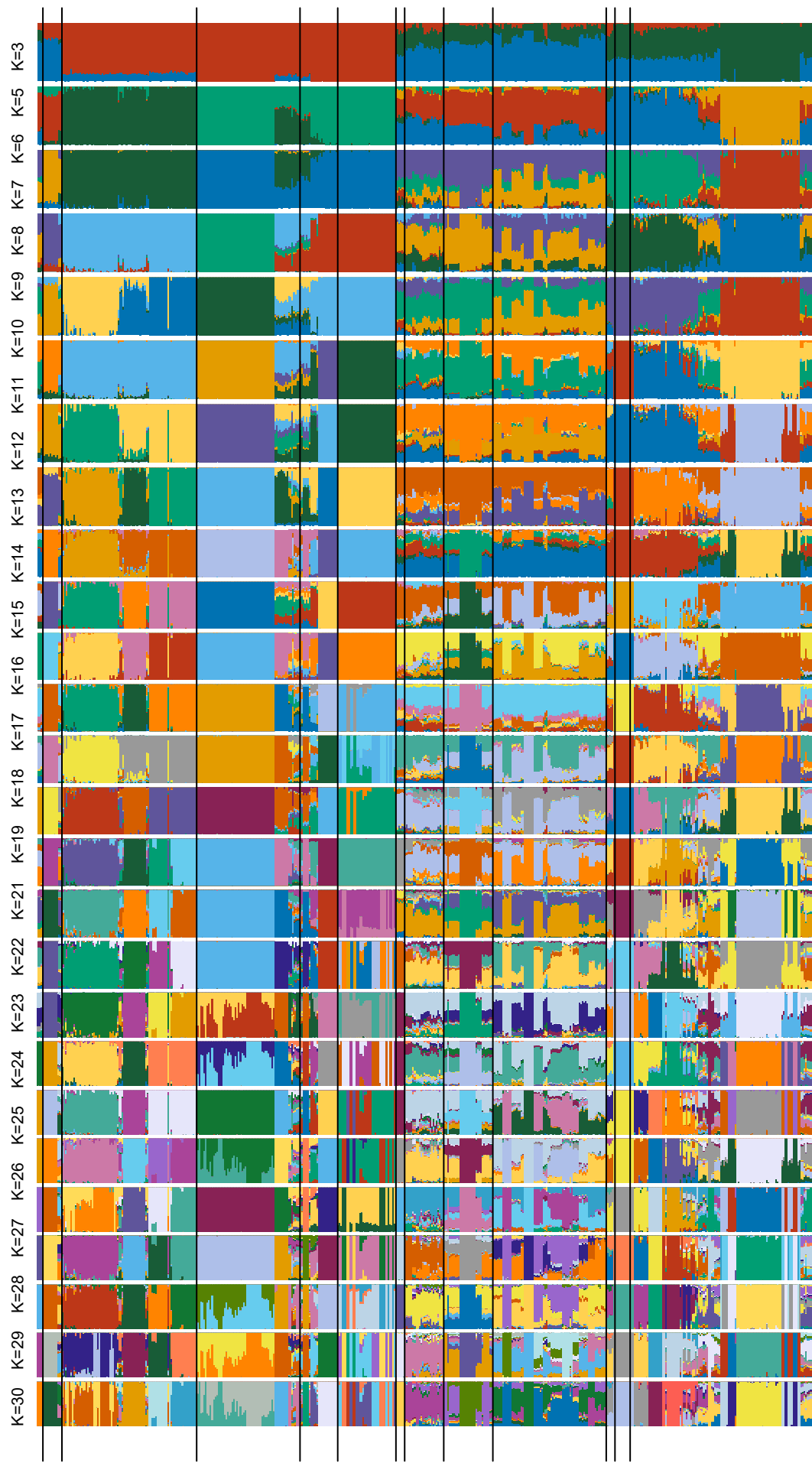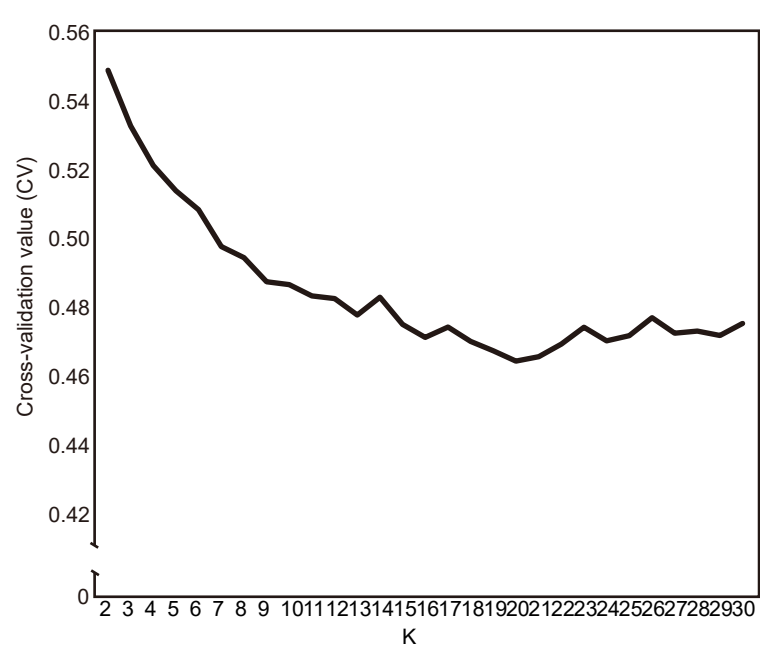

Supplement: Supplementary file 5 — Additional file 5: Figure S2. The admixture results in K from 2 to 30, except 2, 4, and 20, and the cross-validation scatter plot. [file 12711_2025_975_MOESM5_ESM.pdf]

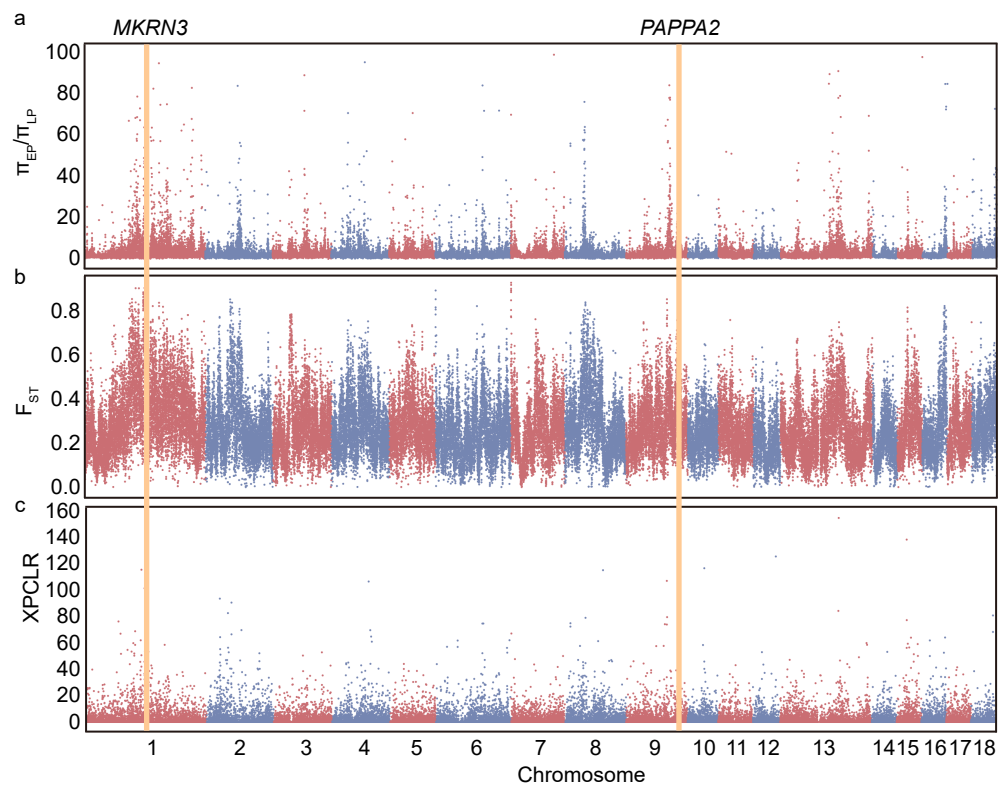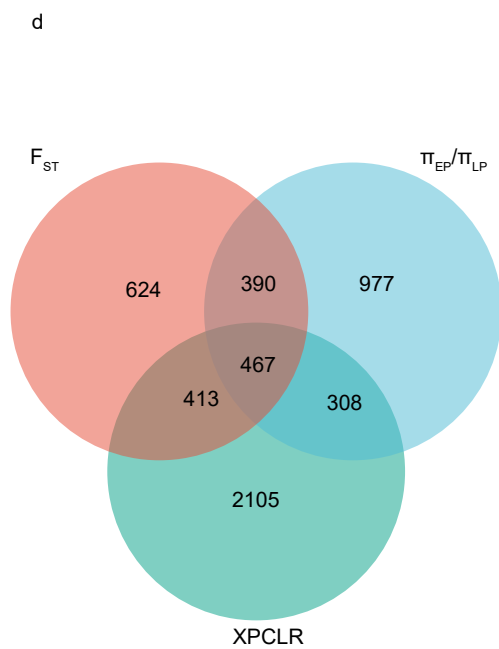

Supplement: Supplementary file 12 — Additional file 12: Figure S3. Venn diagram of three selection signature approaches and shared gene sets. a. The π ratio result is plotted in the Manhattan plot. b. The FST result plotted the Manhattan plot. c. The XPCLR result plotted the Manhattan plot. d. The Venn plot showed the sharing gene sets in all of the methods. [file 12711_2025_975_MOESM12_ESM.pdf]

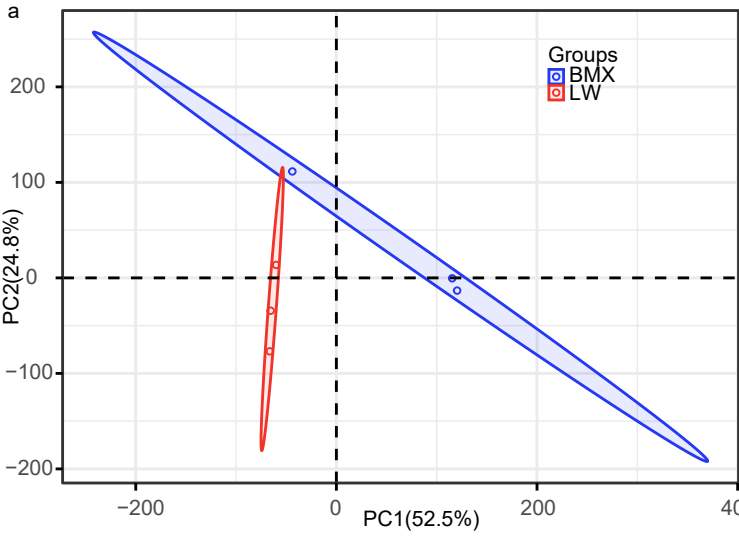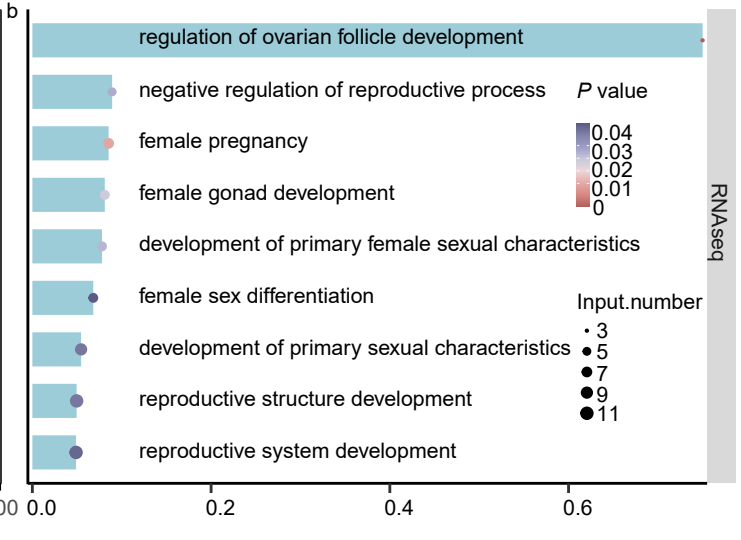

Supplement: Supplementary file 28 — Additional file 28: Figure S4. Transcriptome analysis result. a. PCA plot of transcriptome analysis of pituitary tissues in the LW and BMX groups at 85 days. b. GO enrichment analysis of the DEGs between LW and BMX. [file 12711_2025_975_MOESM28_ESM.pdf]

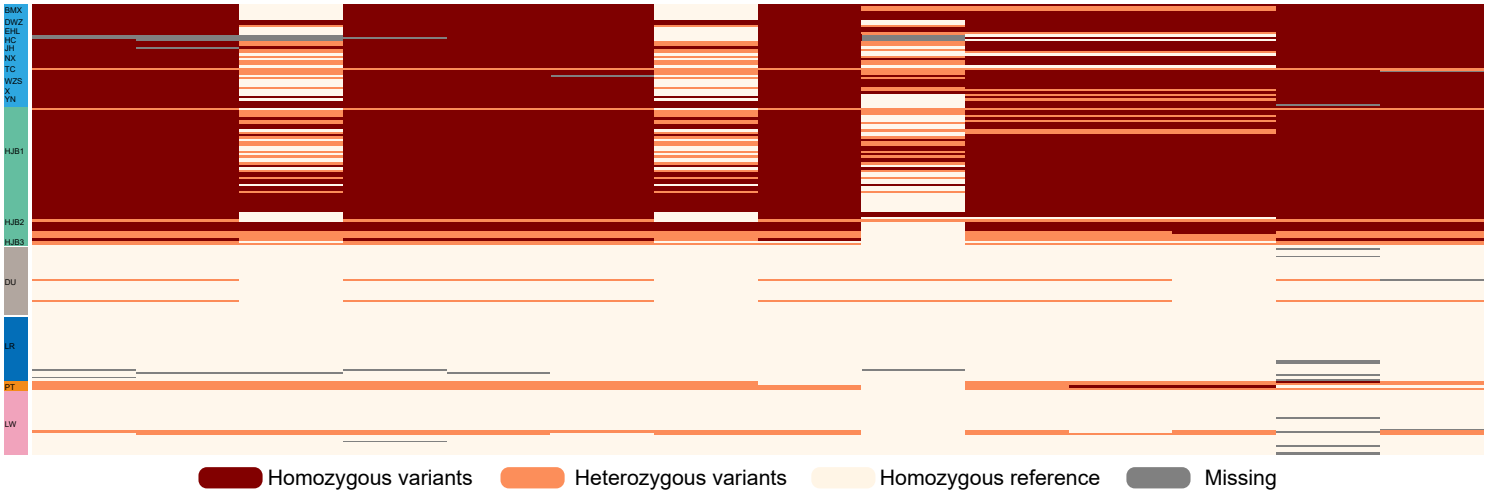

Supplement: Supplementary file 31 — Additional file 31: The haplotype plot of genomic regions at the MKRN3 gene in early and late puberty pigs. [file 12711_2025_975_MOESM31_ESM.pdf]
